# Probing the electrical switching of a memristive optical antenna by STEM EELS

**DOI:** 10.1038/ncomms12162

**Published:** 2016-07-14

**Authors:** David T. Schoen, Aaron L. Holsteen, Mark L. Brongersma

**Affiliations:** 1 Geballe Laboratory for Advanced Materials, Stanford University, Stanford, California 94305-4045, USA

## Abstract

The scaling of active photonic devices to deep-submicron length scales has been hampered by the fundamental diffraction limit and the absence of materials with sufficiently strong electro-optic effects. Plasmonics is providing new opportunities to circumvent this challenge. Here we provide evidence for a solid-state electro-optical switching mechanism that can operate in the visible spectral range with an active volume of less than (5 nm)^3^ or ∼10^−6^ *λ*^3^, comparable to the size of the smallest electronic components. The switching mechanism relies on electrochemically displacing metal atoms inside the nanometre-scale gap to electrically connect two crossed metallic wires forming a cross-point junction. These junctions afford extreme light concentration and display singular optical behaviour upon formation of a conductive channel. The active tuning of plasmonic antennas attached to such junctions is analysed using a combination of electrical and optical measurements as well as electron energy loss spectroscopy in a scanning transmission electron microscope.

## Abstract (web summary)

Scaling of photonic devices requires materials with sufficiently strong elecro-optic effects. Here, Schoen *et al.* demonstrate and analyze single electrically induced switching events that can operate in the visible with a small active volume using electron energy loss in a scanning transmission electron microscope.

## Graphical abstract

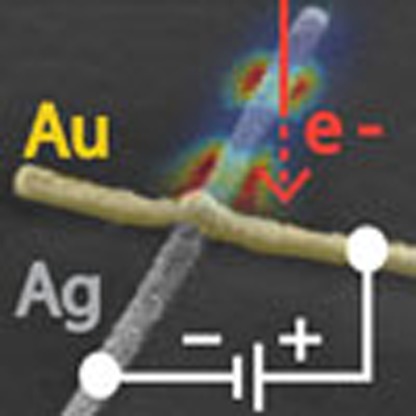

The tremendous benefit derived from scaling device technologies has served as a central driver of science and technology as well as economic growth for many decades. In electronics, nanoscale components are already commonplace and even single-molecule and single-atom devices are being explored^1^,^2^,^3^. In sharp contrast, the miniaturization of optical components to nanometre-sized dimensions has been faced with a seemingly unsurmountable barrier: the optical diffraction limit. Whereas plasmonics^4^ and high-index semiconductor nanostructures^5^ have opened up new ways to manipulate light at the nanoscale, downscaling of active elements to subwavelength footprints remains challenging as one cannot benefit from long interaction lengths to achieve a desired functionality. This is particularly true for a range of technologically relevant electro-optical switching, modulation^6^ and cavity-tuning devices^7^,^8^,^9^, which tend to rely on weak electro-absorption or electro-refraction effects^10^. To address this challenge, researchers are actively exploring a wealth of new materials that display very large changes in their optical properties in response to external stimuli, including tailored quantum wells^11^, phase transformation materials^12^,^13^, organics^14^,^15^, 2D conductors such as graphene^16^,^17^,^18^,^19^ and epsilon-near-zero materials^20^,^21^,^22^. New device physics concepts employing plasmonics^23^,^24^,^25^ or optically resonant structures^26^ are also being investigated. This general approach to scale active components has been quite successful for the recent development of electrically driven nanolasers and spasers^27^,^28^. However, despite many efforts the smallest, power-efficient electro-optical devices have remained micron-scale or larger^29^,^30^. One key reason for this is that all of the traditional switching/tuning strategies fundamentally rely on the interaction of light with mobile charges (electrons or holes) and electrical gating only affords relatively small changes in their volume density (∼10^21^ cm^−3^) before electric fields reach unsustainable levels and electrical breakdown occurs. In this work, we capitalize on the massive changes in mobile carrier density (∼10^23^ cm^−3^) that can be achieved without a net charge build-up by physically moving metal atoms via an electrochemical process. In our switch-design, these atoms are moved into the nanometre-scale gap to create a tiny conductive filament between two crossed metallic wires forming a cross-point junction. As the illumination of such junctions results in effective light concentration inside the gap via the excitation of surface plasmons, its optical properties are very sensitive to any changes in material properties in the gap. This then facilitates the realization of a new type of solid-state electro-optical switching mechanism that can operate in the visible and near-infrared spectral range with an active volume less than (5 nm)^3^ or about 10^−6^ *λ*^3^, that is, comparable to the size of smallest active electronic components. Whereas we do not visualize the very small filament in our study, we do provide several pieces of indirect experimental evidence that are consistent with the formation of such a filament.

Cross-point junctions are among the most compact and densely integrated electronic devices at 10^11^ devices per square centimetre^31^. This architecture has already seen widespread use in molecular electronics, nonvolatile memory^32^,^33^ and most recently memristive logic elements have been used for computation^34^,^35^,^36^. Most of these devices rely on the electric-field induced transport of ionic species to reversibly grow and dissolve a nanoscale conductive filament. This filament has recently been observed to be just a few nanometres in size^37^ and its tip is scalable to the single-atom level^38^. Because the total volume of this filament is extremely small, the charge transport required to form it is also very small. This facilitates high switching speeds (<1 ns or >1 GHz) and very low switching energy (<100 fJ)^39^. These desirable features are combined with a high retention time (years) and impressive endurance (>10^12^ cycles). Whereas these desirable traits already opened up a wide range of electronics applications, applications in photonics have not yet been reported.

In this work, we demonstrate and analyse single electrically induced switching events in detail using a combination of electrical and optical measurements as well as electron energy loss spectroscopy (EELS) in a scanning transmission electron microscope (STEM). We show that a switching event affords significant control over the spatial and spectral properties of the local density of states (LDOS). This study complements two other recent studies on deep-subwavelength, electrically-controlled optical switches that were published during the review of this work^40^,^41^.

## Results

### Basic properties of a memristive optical antenna

**Figure 1:**
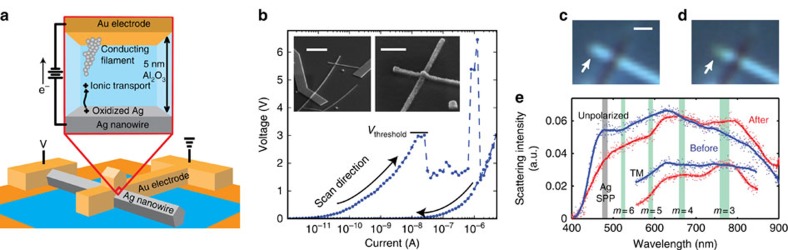
Electrical and optical switching properties of a memristive optical antenna. (**a**) Schematic of the device with a metallic cross-point junction made from a lithographically defined Au electrode, an Al_2_O_3_ insulating spacer, and a chemically synthesized silver nanowire. The zoom-in illustrates the proposed mechanism of operation involving the formation of a conductive metallic filament in the cross-bar junction. (**b**) A typical current-scan on a cross-point junction resulting in dielectric breakdown via the formation of a metallic filament. The formation of the filament results in a state of the device with a significantly lower resistance. Inset: scanning electron micrographs of the tested cross-point junction and a zoom-in with scale bars of 2 μm and 600 nm respectively (**c**) Bright-field optical microscopy images showing the change in the scattering response before (blue) and (**d**) after (red) electrical switching under unpolarized, white-light illumination. Scale bar, 500 nm. (**e**) Confocally detected white-light-scattering spectra taken from the cross-point junction device shown in **c** and **d**. Both unpolarized and transverse magnetic (TM)-polarized spectra are shown. The silver column shows the anticipated spectral location for the localized surface plasmon resonance supported by a subwavelength-diameter silver nanowire. The green columns correspond to the predicted location of different order (*m*=3, 4, 5, 6) Fabry-Pérot (FP) resonances of surface plasmon polaritons oscillating between the Ag nanowire truncation and cross-point. The width of the green beams corresponds to the expected change in resonance frequency on switching the junction from an FP resonance model.

Figure 1a shows a schematic of a metallic cross-point junction designed to elicit a significant change in an optical scattering response by moving about 10^2^–10^3^ metal atoms in and out of the junction region. The design is inspired by a conventional electrochemical memory device in which the active electrode is a chemically synthesized silver (Ag) nanowire, the ion conducting layer is a 5-nm-thick insulating aluminum oxide (Al_2_O_3_) layer grown by atomic layer deposition (ALD), and the inert counter electrode is an evaporated gold stripe defined by electron beam lithography (EBL)^34^,^35^. The application of a positive bias is known to cause oxidation of the Ag electrode, which drives out Ag cations. The cations subsequently migrate to the inert counter electrode where they are reduced to form a metal Ag filament that grows back towards the Ag electrode. Previous research on Au–Al_2_O_3_–Ag junctions suggested that metallic silver filaments of just a few nanometres in diametre can form in this materials system^42^. The inset to Fig. 1b shows a scanning electron microscopy (SEM) image of a tested cross-point junction and a zoom-in of this junction. To carefully study the switching event, we induced filament growth by sourcing a current through the junction and ramping up the current logarithmically from the pA level to 5 μA over several seconds. When electrical breakdown was achieved, a sudden kink in the IV can be observed. For the fabricated junctions, this typically occurred at a threshold voltage around 3 V (Fig. 1b). After the initial breakdown event takes place, the IV characteristics vary stochastically. This behaviour is often referred to in nonvolatile memory studies as the ‘forming' stage^36^, in which the filament is created through Ag ion migration. After the filament is formed, the device follows a different return trajectory with a much lower resistance. In this particular device the DC resistance dropped by more than three orders of magnitude. The DC resistance is checked by a voltage-sourced IV scan with a small current compliance of 1 nA to avoid making changes to the junction. The measured resistance before breakdown is greater than 1 GΩ, and after breakdown is around 6 × 10^2^ kΩ. This resistance is much larger than that of the resistance of the metallic leads leading up to the junction of ∼65 Ω, as well as that of a single-atomic point contact *R*_0_=(2*e*^2^/*h*)^−1^≈12.9 kΩ (ref. 43). This is consistent with previous work that indicates the formation of a filament can abruptly be terminated when using a small current compliance during the growth phase. This is attributed to the rapidly decreasing electronic tunneling resistance from the filament to the electrode with decreasing gap-size that switches off the ionic current flow and thus the filament growth. This results in a final, switched device state for which the total resistance is composed of a larger tunnel resistance in series with a smaller filament resistance^44^,^45^.

In general, the optical detection of a metallic filament that is just a few nanometres in size would be extremely challenging. However, unpolarized white-light-scattering images taken from several devices show how the Ag stub-antenna connected to the cross-point junction can be used to gain information on changes occurring inside the junction. These images show a clear change in the light-scattering response on dielectric breakdown in the junction. Figure 1c,d shows an example where the region near the termination of the Ag nanowire changes in colour from blue to green right when its resistive state is switched. This change occurs without an observable modification in the junction geometry in the optical or SEM images. The spectral changes were confirmed and quantified by taking confocal, white-light-scattering spectra collected from this area. The overall scattering spectrum shows a noticeable redshift on switching the device, suggesting that the change in the conductive state of the junction plays an important role in the resonant scattering behaviour of the antenna (Fig. 1e). Multiple electronic and optical switching events are discussed in Supplementary Fig. 1. In this work, we will focus on a detailed analysis of the changes in the optical properties that occur with the initial switching event from the highly resistive to the more conductive state of the junction.

In analysing the structure of the multi-peaked scattering spectrum before switching (blue curve), it is clear that several optical resonances must be contributing to the scattered light signal in the far-field. One can clearly identify the presence of the localized surface plasmon resonance of the Ag nanowire peaked at 480 nm (ref. 46). In addition, one would expect to see a series of Fabry–Pérot type resonances for surface plasmon polaritons (SPPs) that can propagate back and forth between the Ag nanowire truncation and the cross-point junction^47^,^48^,^49^,^50^. When the length of this nanowire section is taken to be *L*, one would expect resonances to occur whenever the SPP roundtrip phase *φ*_RT_=2*k*_SPP_*L*+*φ*_CR_+*φ*_TR_ equals an integer *m* times 2*π*. Here *k*_SPP_ is the SPP propagation constant and *φ*_CR_ and *φ*_TR_ are the reflection phases that the SPP incurs in reflecting from the nanowire crossing point and the nanowire truncation, respectively^48^. The value of *m* is the mode number and indicates the number of antinodes in the oscillating SPP current. Using the experimentally derived SPP dispersion relation and reflection phases for this type and size of Ag nanowire from a previous work^50^, we calculated the spectral locations where low-order Fabry–Pérot type resonances are expected (green columns in Fig. 1e). On switching the device, one could expect all of the Fabry–Pérot resonances to redshift by several tens of nanometres as the electric connection causes an ∼*π*/4 increase in *φ*_CR,_ as determined from a previous work in which two metallic wires were mechanically placed in electrical contact^50^,^51^. This is consistent with the overall redshift of the spectrum.

As the SPP resonances are longitudinal resonances, one would expect the maximum impact of these resonances on the scattering spectra collected for transverse magnetic (TM) polarized light for which the electric field is oriented along the length of the Ag nanowire. Furthermore, the lowest-order resonances occurring at the longer wavelengths are expected to be strongest due to the longer SPP propagation length at longer wavelengths^49^,^50^. The inset to Fig. 1e shows the TM spectra near the expected location of the *m*=3 and *m*=4 resonance. Indeed, two red-shifting peaks are seen in this spectral range. The observed changes in the far-field light-scattering measurements provide direct evidence for the significant modification in the scattering properties. The observed changes also appear to be in reasonable qualitative agreement with the changes expected from a basic Fabry–Pérot-resonance model. However, the presence of a plurality of overlapping far-field scattering resonances makes it a very challenging (if not impossible) task to construct and verify an exact quantitative model for the scattering spectrum. This is further complicated by the fact that the excitation and collection efficiencies for the different resonances are quite sensitive to the excitation conditions and exact device geometry.

### Switching characterization with STEM EELS

To get around the challenges associated with analysing the far-field light-scattering spectra, we use a STEM capable of creating high-spatial-resolution maps of both the morphology of the device structure as well as the LDOS by performing electron energy loss spectroscopy EELS^49^,^50^,^52^,^53^,^54^. This requires that the device structures be fabricated on 15-nm-thick, free-standing electron-transparent silicon nitride membranes, here obtained from Ted Pella (Supplementary Figs 2 and 3). This procedure facilitates an effective visualization of the optical modes supported by the cross-point devices. To our knowledge, this work also constitutes the first EELS study on an active device in which spectral and spatial modifications of the optical properties (LDOS) were followed to analyse its operation.

**Figure 2:**
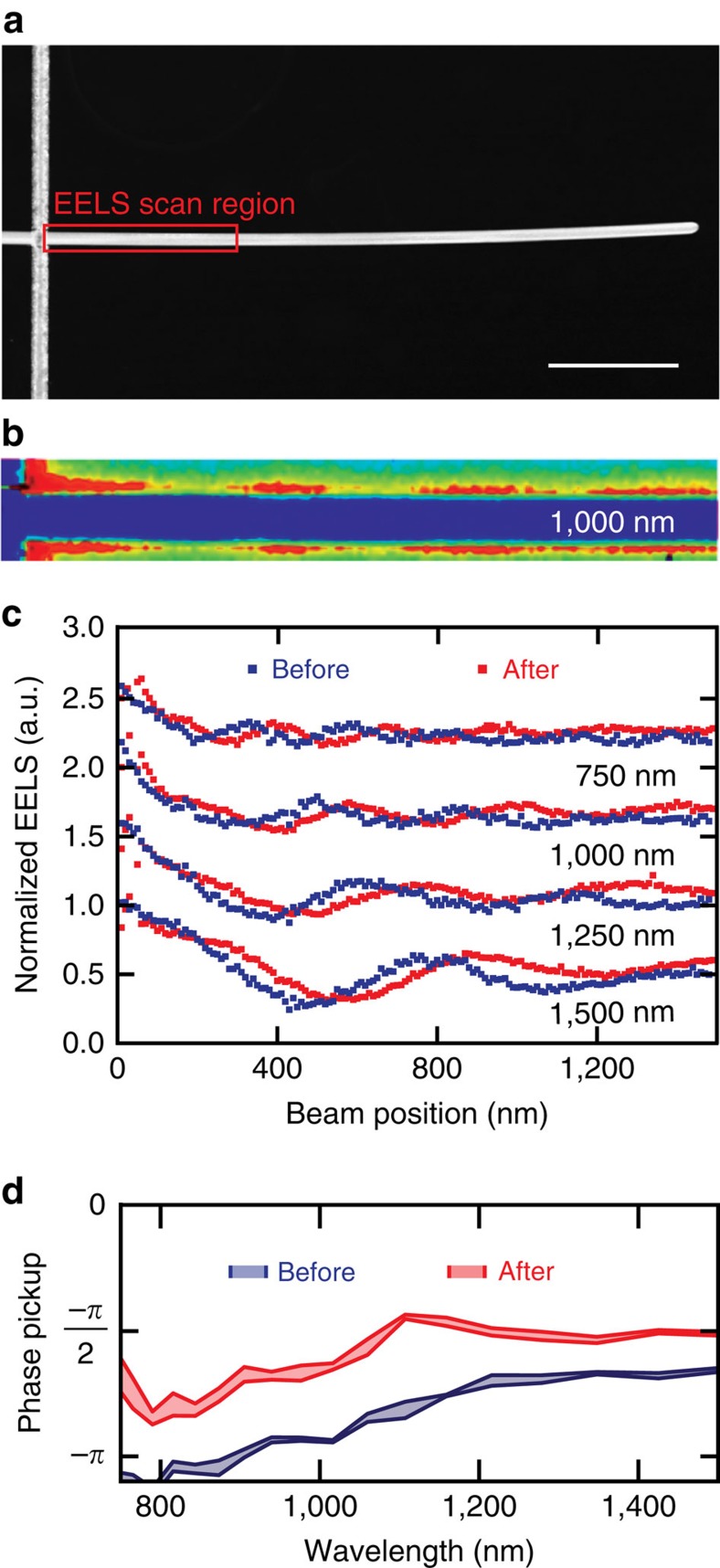
Analysing the changes in the local density of optical states on switching. (**a**) STEM image of a cross-point junction device from which an Ag nanowire extends well beyond the junction. Scale bar, 1 μm. (**b**) STEM EELS map taken from the area outline in **a** and for an energy corresponding to a free-space wavelength of 1,000 nm. (**c**) Line profiles of the EELS signal along the Ag nanowire as extracted from STEM maps as shown in **b**. Line profiles are shown at several electron energy losses that correspond to free-space wavelengths in the range from 750 nm to 1,500 nm. Line scans before (blue) and after (red) show the spatial shift in the locations of the antinodes as result of the electrical switching event. (**d**) The changes in the reflection phase of SPPs from the cross-point junction on switching. The values are extracted from EELS line scans as shown in **c**. The width of the lines indicates the error in the fits to the experimental EELS data.

To investigate the impact of the filament growth on *φ*_CR_, we first analyse a cross-point junction device with an Ag nanowire that is quite long compared with the SPP decay length. Figure 2a shows a STEM image of the junction and Fig. 2b shows a spatial map of the EELS signal before switching for an energy of 1.24 eV, corresponding to a free-space wavelength of 1,000 nm. Such maps provide direct information on the spatial distribution of the LDOS as the loss probability for electrons passing by the antenna is exactly proportional to the LDOS projected along the electron beam trajectory^54^. A series of nodes and antinodes can be seen close to the junction indicating a periodic variation in the LDOS at a spatial scale that is equal to half the surface plasmon wavelength. The variations are reminiscent of Friedel oscillations in the local electronic density of states near the surface of a metal object^55^.

To further analyse this data, we integrated the EELS signal perpendicular to the nanowire axis to generate a line-scan. This line-scan and those for other energy loss values are shown in red in Fig. 2c. Previously, it was shown how such line scans can be analysed to extract the real and imaginary parts of the SPP dispersion as well as the reflection phases for SPPs reflecting from cavity truncations^50^. When we extract the magnitude of *φ*_CR_ from line scans at different energy loss values, we can see that large values close to −*π* are achieved, especially for the short wavelengths for which the SPPs are most confined. The red line scans in Fig. 2c represent the data after the device is taken out of the TEM, the electrical state of the device is changed from a high to a low-resistance state and immediately brought back into the TEM. A significant shift in the antinode position relative to the junction location at *x*=0 can be observed. When we extract the phase pickup on reflection from this data, we can see that a nearly constant shift of *π*/4 is realized across the observed wavelength range.

At first sight, the *π*/4 shift in the reflection phase may appear to be unexpectedly large for the nanoscopic change in the geometry. However, changes of this magnitude can be understood in light of a recent observation that nearly touching metallic nanostructures feature a singular optical response in the limit they become (electrically) touching^56^. At this point, the flow of charge induced by a plasmonic excitation in the junction can markedly change as transfer of net, mobile charge between the electrodes becomes possible. This in turn can result in easily observable changes in the optical response and the supported plasmonic modes^50^,^56^. Recently, detailed TEM EELS studies on conductively coupled metal dimer structures have also visualized such behaviour in a convincing manner^57^,^58^. These large changes in optical response can also be understood from circuit models that describe the optical behaviour of two closely spaced metal objects in which a load is placed in the gap. Within the context of such a model, the impedance of the load *Z*_load_ can be treated as a parallel circuit composed of an inductive impedance *Z*_f_ formed by the metallic filament and a capacitive impedance Z_gap_ formed by gap between the two electrodes spaced by Al_2_O_3_. Significant changes in the response are expected when the filament grows and *Z*_f_ approaches *Z*_gap_^59^,^60^,^61^. This geometry has recently stimulated significant interest in the fundamental question whether quantum mechanical tunneling processes between metallic structures can induce these significant changes in the optical response^62^,^63^,^64^,^65^. This work provides further support for this hypothesis. More importantly from a practical perspective, it shows how this unique singular optical behaviour can be harnessed to realize the smallest possible active photonic device technology.

### Tuning the optical resonances of a memristive antenna

**Figure 3:**
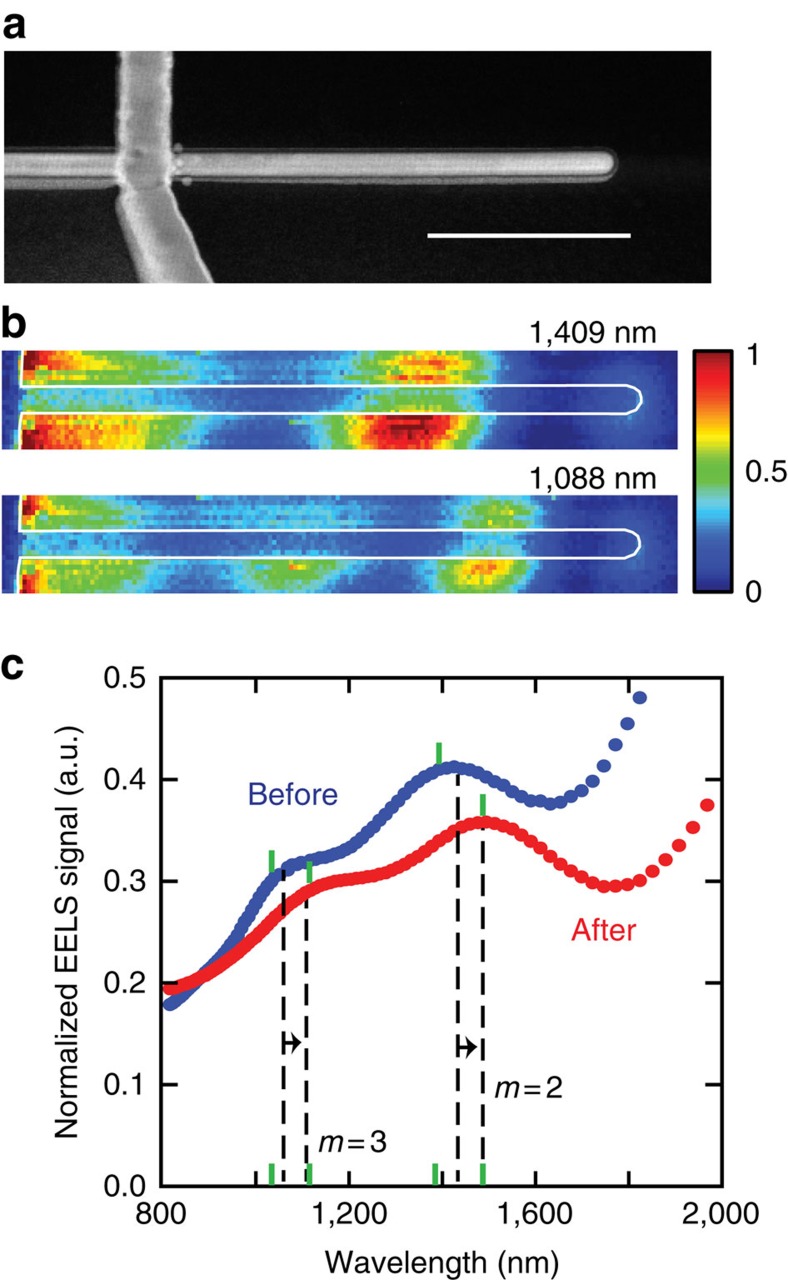
Electrical tuning of a memristive antenna as verified using EELS maps and EELS spectra. (**a**) STEM image of a memristive optical antenna after breakdown showing that the overall morphology is intact and that the Au and Ag lines remain morphologically distinct. Scale bar, 400 nm. (**b**) STEM EELS maps for the second and third-order resonant modes after switching (**c**) STEM EELS spectra averaged over an area including the antenna showing a redshift in resonant frequencies before and after breakdown. The dashed lines indicate the spectral locations of the Fabry–Pérot resonances for this antenna and the green marks show the deconvolved locations of the EELS plasmon resonances.

Next, we discuss the opportunity to tune the resonant response of a finite-sized Ag nanowire antenna. Figure 3a shows an STEM image of a device with an 1150-nm section of Ag nanowire extending from the cross-point. Figure 3b shows EELS maps for the second (*m*=2) and third (*m*=3) order resonant plasmonic modes of the Ag antenna at energies of 0.88 eV (*λ*_0_=1,409 nm) and 1.14 eV (*λ*_0_=1,088 nm), respectively. The ability to spatially map the LDOS at different energy loss values enables one to assign and separately study the different Fabry–Pérot resonant modes supported by the antenna structure. This shows one of the major benefits over far-field optical light-scattering studies where it is very challenging to separate and quantify contributions from different optical modes to the measured signal. From the Fabry–Pérot resonance condition it is clear that for these low-order modes the reflection phases can be comparable to the propagation phase. As such, the easily observable, electrically induced changes in *φ*_CR_ seen in Fig. 2d can have a very large impact on its resonant scattering properties. The results of the electrical switching on this device are included in Supplementary Fig. 4, but the response was qualitatively similar to the results in Fig. 1b. We can see that both of the observed resonances redshift in spectral position by 96 and 78 nm for the second and third-order peaks, respectively. The spectral locations of the SP plasmon peaks in the EELS maps were obtained by deconvolving the spectrum using a Voigt profile with three Lorentzian resonance peaks associated with the first three plasmonic resonant modes^66^. These redshifts are of a similar magnitude as those observed in the white-light-scattering spectra of Fig. 1e after dielectric breakdown. The theoretical values predicted by the Fabry–Pérot model using the reflection phases given in Fig. 2d predict a 65- and a 20-nm redshift in the second and third-order resonances of a silver nanowire antenna 1,090 nm in length, respectively, that is, of a similar magnitude as those found in the experiments. This is a remarkable shift considering that no change to the geometry of the device could be observed by STEM imaging after the breakdown event.

## Discussion

We have demonstrated a solid-state electro-optical switching mechanism that can operate in the visible spectral range with an active volume of less than (5 nm)^3^ or ∼10^−6^ *λ*^3^. This size is comparable to that of the smallest electronic components and thus could facilitate a more seamless integration of active electronic and optical components. The possibility to electrically induce significant changes in the optical response of integrated optical antennas can be applied in a wide range of practical applications in which tuning of light emission and absorption by quantum emitters is desired. These antennas in single or dense arrays that form metamaterials can also be used to manipulate the flow of light in densely packed cross-point devices. More fundamentally, the experiments suggest a fundamentally new pathway to switch solid-state antenna devices and pave the way for the realization of optoelectronic modulators and cavity-tuning devices with an unprecedented footprint below 0.01 μm^2^.

## Methods

### Device fabrication

Fabrication of the memristive optical antennas was accomplished by a combination of bottom–up and top–down nanofabrication approaches to fabricate the devices on thin nitride membranes (Supplementary Fig. 2). First a set of electron transparent substrates was prepared using commercially obtained silicon nitride membranes (15-nm thick, Ted Pella). Such thin membranes are required to maintain a good energy resolution during EELS. Next a set of dielectric pads were processed using EBL to pattern spin-on glass. Then a series of large gold contact pads (50-nm thick) was placed by EBL and DC sputtering. Silver nanowires grown chemically by the polyol procedure were randomly dispersed on the prepared substrate^67^. The nanowires were coated by atomic layer deposition with ∼100 cycles of Al_2_O_3_, using trimethyl aluminum and water vapour as precursor gasses. An image of a silver nanowire coated with 120 cycles of ALD is shown in Supplementary Fig. 5, showing that the Al_2_O_3_ grows conformally on the silver nanowires. These nanowires were located with the SEM, and smaller gold electrodes were fabricated to connect the silver nanowires to the large contact pads. To apply bias to only one gold/Al_2_O_3_/silver junction, it was necessary to connect electrically one of the two gold lines to the metal core of the Al_2_O_3_ coated silver nanowire. This was accomplished by the deposition of platinum using a focused ion beam, where the gallium ion beam used to assist in the platinum deposition damaged the dielectric and lead to a short, which is then coated with the conductive platinum (Supplementary Fig. 3).

### Device characterization

Light-scattering measurements on the silver antennas protruding from the junctions were carried out using a confocal optical microscope coupled to a true-colour CCD (Nikon) and a spectrometre (Spectra Physics) with a cooled CCD (Princeton Instruments, Pixis 1,024). The silver nanowire devices were illuminated in the bright-field objective (Nikon NA0.9, × 100) with white light from a halogen lamp. The light from the silver nanowire antenna was confocally selected and sent to the spectrometre to acquire quantitative spectral information. The spectral data were normalized by subtracting the spectrum backscattered from the air–glass interface from the spectrum of the antenna, then dividing by the lamp spectrum. The spatial resolution of the confocal collection scheme was ∼1 μm. The estimated resistance of the metallic leads and the nanowire of the cross-point junction where calculated using COMSOL Multiphysics using the electric currents module for a device of the same dimensions as the device measured in the optical and STEM EELS experiments.

### Data availability

All relevant data are available from the authors on request.

## Additional information

**How to cite this article:** Schoen, D. T. *et al.* Probing the electrical switching of a memristive optical antenna by STEM EELS. *Nat. Commun.* 7:12162 doi: 10.1038/ncomms12162 (2016).

## Supplementary Material

Supplementary Information Supplementary Figures 1-5

## References

[b1] Reed M. A. Conductance of a molecular junction. Science 278, 252–254 (1997).

[b2] Fuechsle M. *et al.* A single-atom transistor. Nat. Nanotechnol. 7, 242–246 (2012). 22343383 10.1038/nnano.2012.21

[b3] Strukov D. B., Snider G. S., Stewart D. R. & Williams R. S. The missing memristor found. Nature 453, 80–83 (2008). 18451858 10.1038/nature06932

[b4] Brongersma M. L. Introductory lecture: nanoplasmonics. Faraday Discuss. 178, 9–36 (2015). 25968246 10.1039/c5fd90020d

[b5] Brongersma M. L., Cui Y. & Fan S. Light management for photovoltaics using high-index nanostructures. Nat. Mater. 13, 451–460 (2014). 24751773 10.1038/nmat3921

[b6] Reed G., Mashanovich G., Gardes F. & Thomson D. Silicon optical modulators. Nat. Photon. 4, 518–526 (2010).

[b7] Perney N. M. B. *et al.* Tuning localized plasmon cavities for optimized surface-enhanced Raman scattering. Phys. Rev. B. 76, 035426 (2007).

[b8] Tam F., Goodrich P. G., Johnson R. B. & Halas J. N. Plasmonic enhancement of molecular fluorescence. Nano Lett. 7, 496–501 (2007). 17256995 10.1021/nl062901x

[b9] Tanabe T., Notomi M., Taniyama H. & Kuramochi E. Dynamic release of trapped light from an ultrahigh-Q nanocavity via adiabatic frequency tuning. Phys. Rev. Lett. 102, 043907 (2009). 19257423 10.1103/PhysRevLett.102.043907

[b10] Soref R. & Bennett B. Electrooptical effects in silicon. IEEE J. Quantum Electron. 23, 123–129 (1987).

[b11] Kuo Y.-H. *et al.* Strong quantum-confined Stark effect in germanium quantum-well structures on silicon. Nature 437, 1334–1336 (2005). 16251959 10.1038/nature04204

[b12] Gholipour B., Zhang J., MacDonald K. F., Hewak D. W. & Zheludev N. I. An all-optical, non-volatile, bidirectional, phase-change meta-switch. Adv. Mater. 25, 3050–3054 (2013). 23625824 10.1002/adma.201300588

[b13] Schoen D. T., Xie C. & Cui Y. Electrical switching and phase transformation in silver selenide nanowires. J. Am. Chem. Soc. 129, 4116–4117 (2007). 17367137 10.1021/ja068365s

[b14] Koos C. *et al.* All-optical high-speed signal processing with silicon–organic hybrid slot waveguides. Nat. Photon. 3, 216–219 (2009).

[b15] Melikyan A. *et al.* High-speed plasmonic phase modulators. Nat. Photon. 8, 229–233 (2014).

[b16] Liu M. *et al.* A graphene-based broadband optical modulator. Nature 474, 64–67 (2011). 21552277 10.1038/nature10067

[b17] Li W. *et al.* Ultrafast all-optical graphene modulator. Nano Lett. 14, 955–959 (2014). 24397481 10.1021/nl404356t

[b18] Youngblood N., Anugrah Y., Ma R., Koester S. J. & Li M. Multifunctional graphene optical modulator and photodetector integrated on silicon waveguides. Nano Lett. 14, 2741–2746 (2014). 24734877 10.1021/nl500712u

[b19] Polat E. O. & Kocabas C. Broadband optical modulators based on graphene supercapacitors. Nano Lett. 13, 5851–5857 (2013). 24215484 10.1021/nl402616t

[b20] Feigenbaum E., Diest K. & Atwater H. A. Unity-order index change in transparent conducting oxides at visible frequencies. Nano Lett. 10, 2111–2116 (2010). 20481480 10.1021/nl1006307

[b21] Sorger V. J., Lanzillotti-Kimura N. D., Ma R.-M. & Zhang X. Ultra-compact silicon nanophotonic modulator with broadband response. Nanophotonics 1, 17–22 (2012).

[b22] Vasudev A. P., Kang J.-H., Park J., Liu X. & Brongersma M. L. Electro-optical modulation of a silicon waveguide with an ‘epsilon-near-zero' material. Opt. Express 21, 26387–26397 (2013). 24216861 10.1364/OE.21.026387

[b23] Cai W., White J. S. & Brongersma M. L. Compact, high-speed and power-efficient electrooptic plasmonic modulators. Nano Lett. 9, 4403–4411 (2009). 19827771 10.1021/nl902701b

[b24] Dionne J. A., Diest K., Sweatlock L. A. & Atwater H. A. PlasMOStor: a metal - oxide - si field effect plasmonic modulator. Nano Lett. 9, 897–902 (2009). 19170558 10.1021/nl803868k

[b25] Svitelskiy O. *et al.* A simple cell for the analysis of nanoelectromechanical systems under gas pressure. Rev. Sci. Instrum 79, 093701 (2008). 19044419 10.1063/1.2976675

[b26] Xu Q., Manipatruni S., Schmidt B., Shakya J. & Lipson M. 12.5 Gbit/s silicon micro-ring silicon modulators. Opt. Express 15, 430–436 (2007). 19532260 10.1364/oe.15.000430

[b27] Hill M. T. *et al.* Lasing in metallic-coated nanocavities. Nat. Photon. 1, 589–594 (2007).

[b28] Nezhad M. P. *et al.* Room-temperature subwavelength metallo-dielectric lasers. Nat. Photon. 4, 395–399 (2010).

[b29] Xu Q., Schmidt B., Pradhan S. & Lipson M. Micrometre-scale silicon electro-optic modulator. Nature 435, 325–327 (2005). 15902253 10.1038/nature03569

[b30] Soref R., Fellow L. & Paper I. The past, present, and future of silicon photonics. IEEE J. Sel. Top. Quantum Electron. 12, 1678–1687 (2006).

[b31] Melosh N. A. *et al.* Ultrahigh-density nanowire lattices and circuits. Science 300, 112–115 (2003). 12637672 10.1126/science.1081940

[b32] Waser R. & Aono M. Nanoionics-based resistive switching memories. Nat. Mater. 6, 833–840 (2007). 17972938 10.1038/nmat2023

[b33] Won Seo J. *et al.* Evidence of Al induced conducting filament formation in Al/amorphous silicon/Al resistive switching memory device. Appl. Phys. Lett. 96, 053504 (2010).

[b34] Borghetti J. *et al.* ‘Memristive' switches enable ‘stateful' logic operations via material implication. Nature 464, 873–876 (2010). 20376145 10.1038/nature08940

[b35] Yang J. J. *et al.* Memristive switching mechanism for metal/oxide/metal nanodevices. Nat. Nanotechnol. 3, 429–433 (2008). 18654568 10.1038/nnano.2008.160

[b36] Joshua Yang J. *et al.* The mechanism of electroforming of metal oxide memristive switches. Nanotechnology 20, 215201 (2009). 19423925 10.1088/0957-4484/20/21/215201

[b37] Kwon D.-H. *et al.* Atomic structure of conducting nanofilaments in TiO2 resistive switching memory. Nat. Nanotechnol. 5, 148–153 (2010). 20081847 10.1038/nnano.2009.456

[b38] Terabe K., Hasegawa T., Nakayama T. & Aono M. Quantized conductance atomic switch. Nature 433, 47–50 (2005). 15635405 10.1038/nature03190

[b39] Pickett M. D. & Williams R. S. Sub-100 fJ and sub-nanosecond thermally driven threshold switching in niobium oxide crosspoint nanodevices. Nanotechnology 23, 215202 (2012). 22551985 10.1088/0957-4484/23/21/215202

[b40] Emboras A. *et al.* Atomic scale plasmonic switch. Nano Lett. 16, 709–714 (2016). 26670551 10.1021/acs.nanolett.5b04537

[b41] Di Martino G., Tappertzhofen S., Hofmann S. & Baumberg J. Nanoscale plasmon-enhanced spectroscopy in memristive switches. Small. 12, 1334–1341 (2016). 26756792 10.1002/smll.201503165

[b42] Hickmott T. W. Electroforming and Ohmic contacts in Al-Al_2_O_3_-Ag diodes. J. Appl. Phys. 111, 063708 (2012).

[b43] Krans J. M. *et al.* One-atom point contacts. Phys. Rev. B 48, 14721–14724 (1993). 10.1103/physrevb.48.14721 10007910

[b44] Menzel S., Böttger U. & Waser R. Simulation of multilevel switching in electrochemical metallization memory cells. J. Appl. Phys. 111, 6–11 (2012).

[b45] Tappertzhofen S., Valov I. & Waser R. Quantum conductance and switching kinetics of AgI-based microcrossbar cells. Nanotechnology 23, 145703 (2012). 22434046 10.1088/0957-4484/23/14/145703

[b46] Garnett E. C. *et al.* Self-limited plasmonic welding of silver nanowire junctions. Nat. Mater. 11, 241–249 (2012). 22306769 10.1038/nmat3238

[b47] Novotny L. Effective wavelength scaling for optical antennas. Phys. Rev. Lett. 98, 266802 (2007). 17678117 10.1103/PhysRevLett.98.266802

[b48] Søndergaard T. & Bozhevolnyi S. I. Strip and gap plasmon polariton optical resonators. Phys. Status Solidi 245, 9–19 (2008).

[b49] Rossouw D., Couillard M., Vickery J., Kumacheva E. & Botton G. A. Multipolar plasmonic resonances in silver nanowire antennas imaged with a subnanometer electron probe. Nano Lett. 11, 1499–1504 (2011). 21446717 10.1021/nl200634w

[b50] Schoen D. T., Atre A. C., García-Etxarri A., Dionne J. A. & Brongersma M. L. Probing complex reflection coefficients in one-dimensional surface plasmon polariton waveguides and cavities using STEM EELS. Nano Lett. 15, 120–126 (2015). 25545292 10.1021/nl503179j

[b51] Garnett E. C. *et al.* Self-limited plasmonic welding of silver nanowire junctions. Nat. Mater. 11, 241–249 (2012). 22306769 10.1038/nmat3238

[b52] Nelayah J. *et al.* Mapping surface plasmons on a single metallic nanoparticle. Nat. Phys 3, 348–353 (2007).

[b53] Koh A. L. *et al.* Electron energy-loss spectroscopy (EELS) of surface plasmons in single silver nanoparticles and dimers: Influence of beam damage and mapping of dark modes. ACS Nano 3, 3015–3022 (2009). 19772292 10.1021/nn900922z

[b54] García De Abajo F. J. & Kociak M. Probing the photonic local density of states with electron energy loss spectroscopy. Phys. Rev. Lett. 100, 106804 (2008). 18352220 10.1103/PhysRevLett.100.106804

[b55] Ashcroft N. W. & Mermin N. D. in Ashcroft and Mermin Rinehart and Winston (1976).

[b56] Romero I., Aizpurua J., Bryant G. W. & García De Abajo F. J. Plasmons in nearly touching metallic nanoparticles: singular response in the limit of touching dimers. Opt. Express 14, 9988–9999 (2006). 19529393 10.1364/oe.14.009988

[b57] Alber I. *et al.* Multipole surface plasmon resonances in conductively coupled metal nanowire dimers. ACS Nano 6, 9711–9717 (2012). 23020274 10.1021/nn303149p

[b58] Alber I. *et al.* Visualization of multipolar longitudinal and transversal surface plasmon modes in nanowire dimers. ACS Nano 5, 9845–9853 (2011). 22077953 10.1021/nn2035044

[b59] Schnell M. *et al.* Controlling the near-field oscillations of loaded plasmonic nanoantennas. Nat. Photon. 3, 287–291 (2009).

[b60] Alù A. & Engheta N. Tuning the scattering response of optical nanoantennas with nanocircuit loads. Nat. Photon. 2, 307–310 (2008).

[b61] Alù A. & Engheta N. Input impedance, nanocircuit loading, and radiation tuning of optical nanoantennas. Phys. Rev. Lett. 101, 043901 (2008). 18764328 10.1103/PhysRevLett.101.043901

[b62] Marinica C., Kazansky A. K., Nordlander P., Aizpurua J. & Borisov A. G. Quantum plasmonics: nonlinear effects in the field enhancement of a plasmonic nanoparticle dimer. quantum plasmonics: nonlinear effects in the field enhancement of a plasmonic nanoparticle dimer. Nano Lett. 12, 1333–1339 (2012). 22320125 10.1021/nl300269c

[b63] Zuloaga J., Prodan E. & Nordlander P. Quantum description of the plasmon resonances of a nanoparticle dimer. Nano Lett. 9, 887–891 (2009). 19159319 10.1021/nl803811g

[b64] Savage K. J. *et al.* Revealing the quantum regime in tunnelling plasmonics. Nature 491, 574–577 (2012). 23135399 10.1038/nature11653

[b65] Scholl J. A., García-Etxarri A., Koh A. L. & Dionne J. A. Observation of quantum tunneling between two plasmonic nanoparticles. Nano Lett. 13, 564–569 (2013). 23245286 10.1021/nl304078v

[b66] Bosman M. *et al.* Surface plasmon damping quantified with an electron nanoprobe. Sci. Rep. 3, 1–7 (2013). 10.1038/srep01312 PMC3578264 23425921

[b67] Sun Y. & Xia Y. Large-scale synthesis of uniform silver nanowires. Adv. Mater. 833–837 (2002).

