# Supplementary material for: Probing the electrical switching of a memristive optical antenna by STEM EELS
Source: Nat Commun. 2016 Jul 14;7:12162. doi: 10.1038/ncomms12162 (PMC4947179; doi:10.1038/ncomms12162)
Supplement: Supplementary Information — Supplementary Figures 1-5 [file ncomms12162-s1.pdf]

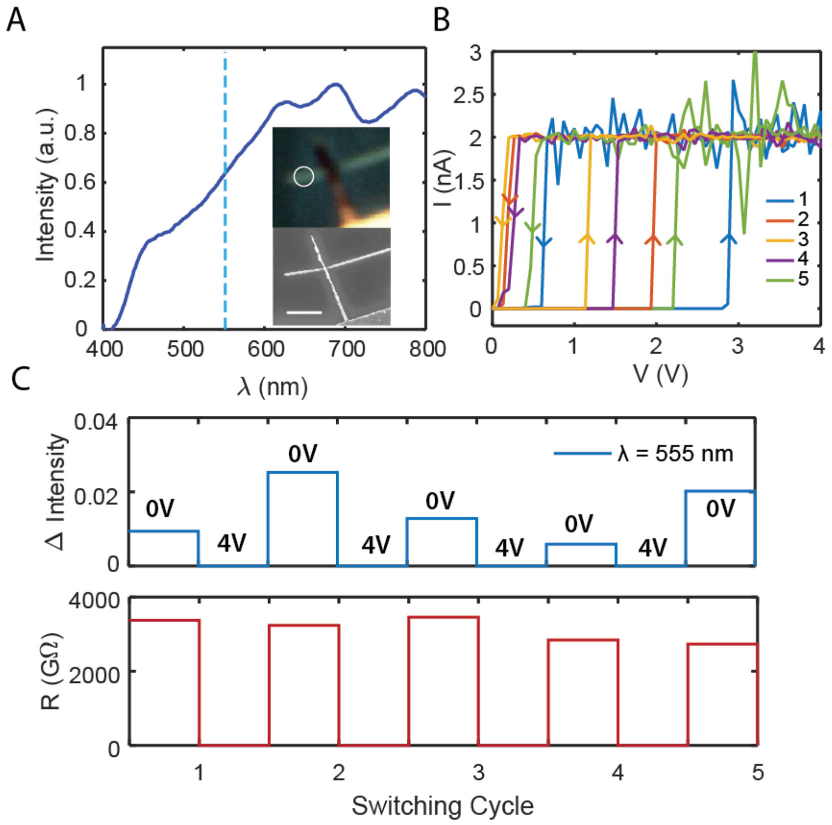

Supplementary Figure 1. Reversible switching behavior (A) Far-field scattering spectra collected by an NA = 0.9 objective of the stub antenna region showing multiple Fabry-Perot fringes. The top inset is the optical image of the device with the circle indicating where the scattered light was confocally collected from. The bottom inset is a SEM image of the device. The scale bar is 1 μm. (B) Five consecutive voltage-sourced switching cycles after the forming stage indicating the reversible electronic switching of the crosspoint junction (C) The upper panel shows the repeatable changes in the scattered light intensity over five switching cycles at a selected wavelength of 550 nm and the lower panel shows the concomitant changes in the measured device resistance during these switching cycles. It is noted that, with the current crosspoint junction design, the changes in the scattered light intensity with the multiple switching cycles are relatively small (on the order of a few %) as compared to the changes seen in the first switching event (see Fig. 1-3 of the main text). This is attributed to the fact that in the first switching event from a resistive to a conductive state a filament is formed that bridges the entire gap between the Au and Ag electrodes. In the subsequent multiple switching events, a more minor, incomplete redissolution of the wire on the nanometer length scale can lead to a dramatic change in the junction resistance based on the exponential dependence of tunnel currents on the electrode spacing. However, the observation of more substantial optical changes could possibly require a complete redissolution of the filament. An optimization of the junction geometry and a decrease of the junction/electrode size can enhance the sensitivity of the optical, light scattering response to the atomic-scale changes to the distribution of atoms in the gap.

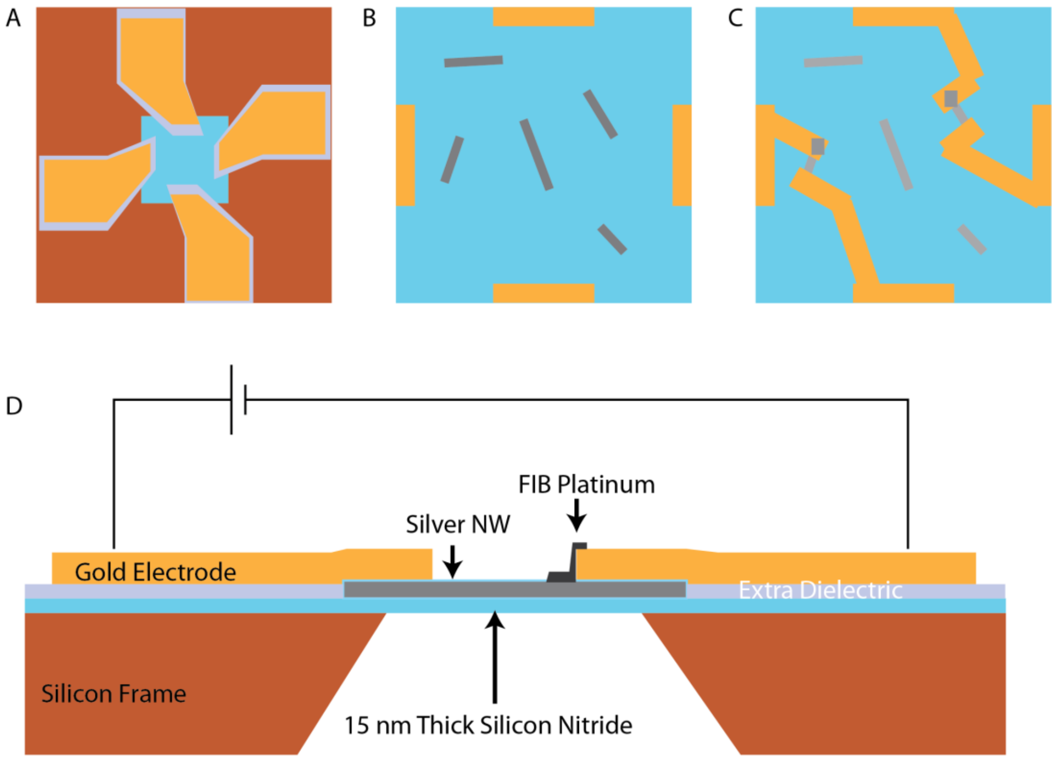

Supplementary Figure 2. Device fabrication procedure. Schematic plan-view sequence of fabrication. (A) Devices are fabricated on prepared 15 nm thick silicon nitride membranes. (B) Silver nanowires are randomly dispersed on the membranes. (C) The nanowires are coated with Al_2O_3, connected by electron beam lithography, and one side is electrically connected to an electrode by ion beam induced deposition of platinum. (D) Schematic cross-section of the full device stack after fabrication.

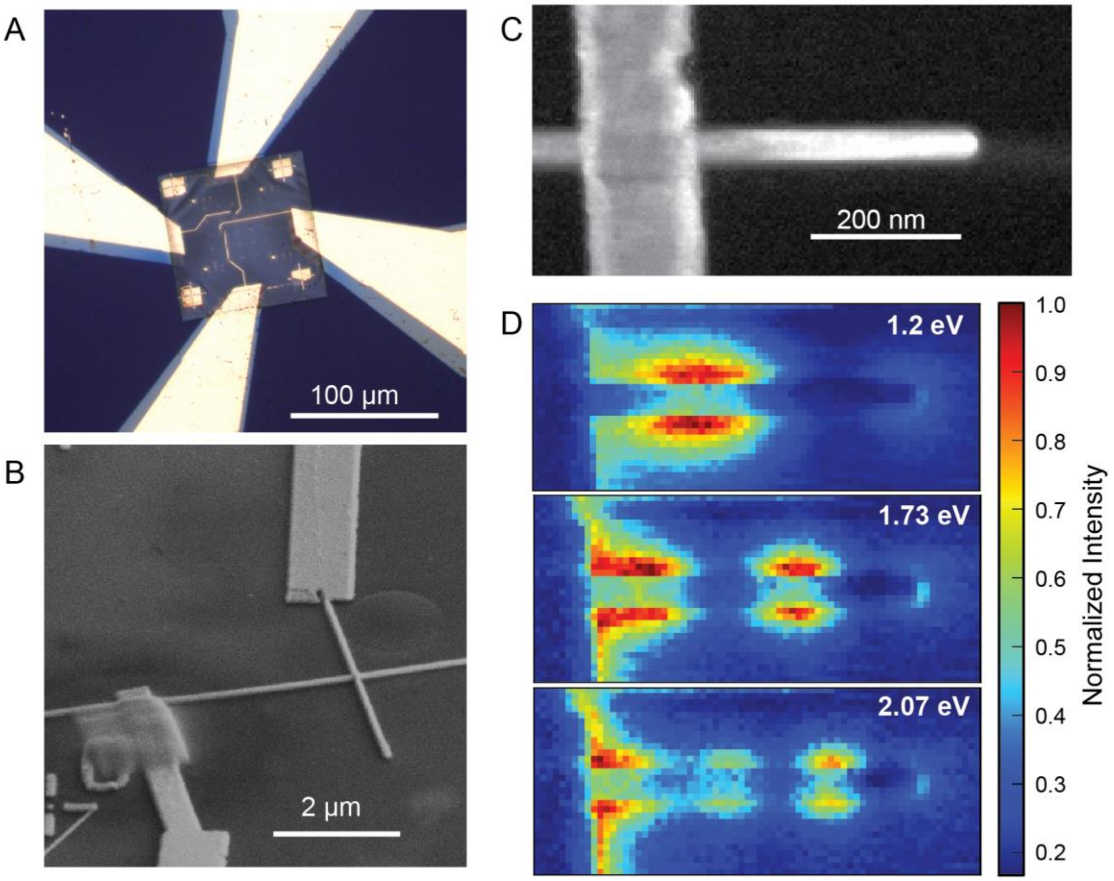

Supplementary Figure 3. MIM device typical of the STEM EELS experimental results (A) Optical micrograph of a MIM device fabricated on a silicon nitride membrane (B) SEM micrograph of a completed device showing the platinum pad making a contact between the silver NW and the gold lead (C) SEM of a silver nanoantenna extending from a contact. (D) The first three resonances of the plasmonic antenna under EELS imaging.

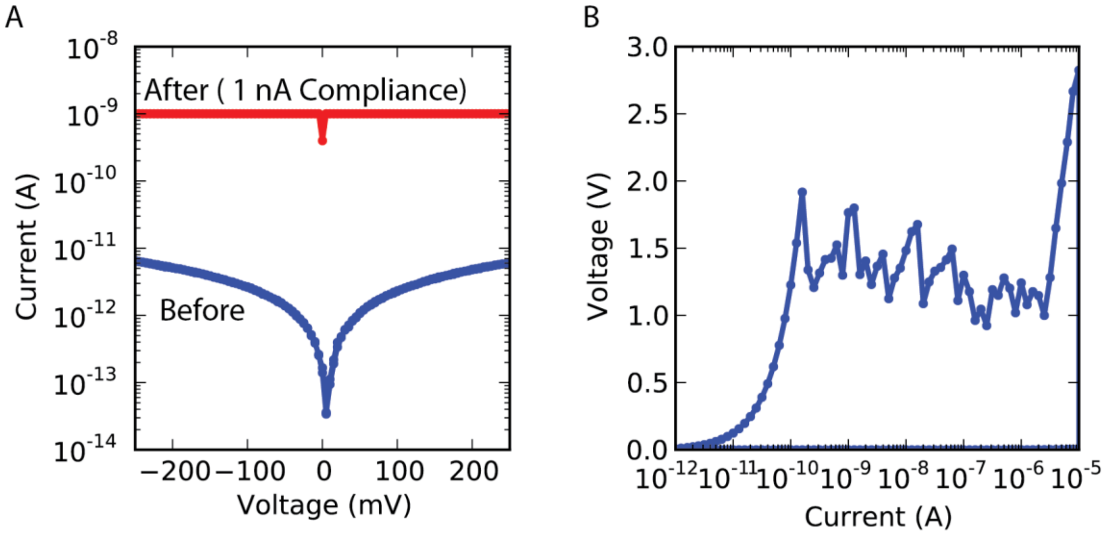

Supplementary Figure 4. Electrical characterization of the MIM device fabricated on a silicon nitride membrane used in Figure 3. (A) DC IV behavior before and after switching. (B) Current sourced switching curve.

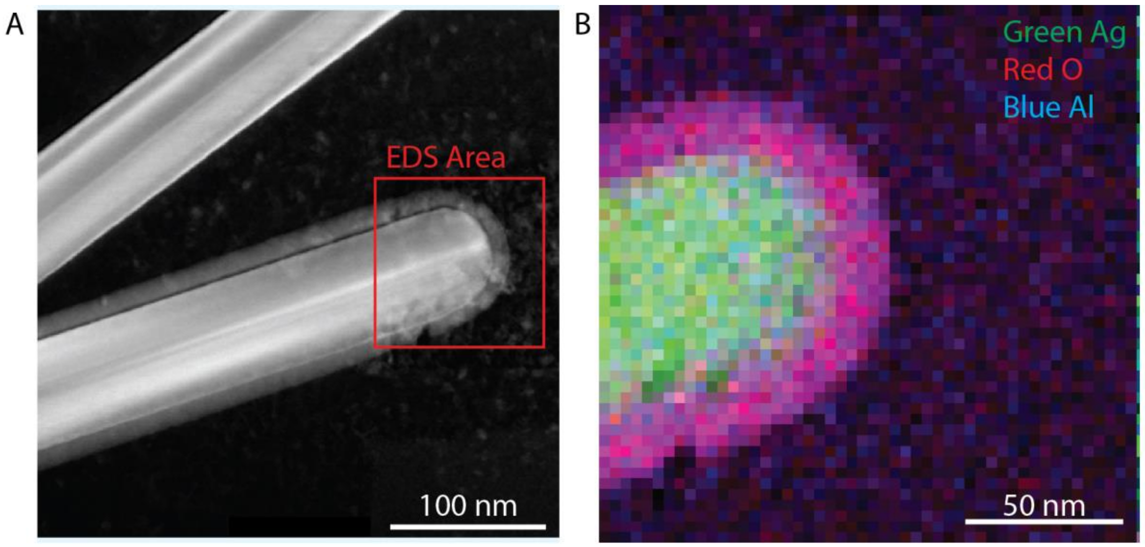

Supplementary Figure 5. Atomic layer deposition of Al_2O_3 on silver nanowires. (A) STEM image of a coated silver nanowire. The second nanowire was deposited after the ALD step and is not coated. (B) EDS map showing silver, oxygen, and aluminum channels.
